# Effects of Three Distinct 2-Week Long Diet Strategies After Transport on Weaned Pigs' Short and Long-Term Welfare Markers, Behaviors, and Microbiota

**DOI:** 10.3389/fvets.2020.00140

**Published:** 2020-03-17

**Authors:** Severine P. Parois, Alan W. Duttlinger, Brian T. Richert, Stephen R. Lindemann, Jay S. Johnson, Jeremy N. Marchant-Forde

**Affiliations:** ^1^PEGASE, Agrocampus Ouest, INRA, Saint-Gilles, France; ^2^USDA-ARS, Livestock Behavior Research Unit, West Lafayette, IN, United States; ^3^Department of Animal Sciences, Purdue University, West Lafayette, IN, United States; ^4^Department of Food Science, Purdue University, West Lafayette, IN, United States

**Keywords:** alternative, L-glutamine, microbiota, pigs, stress, welfare

## Abstract

Alternative feed supplements have shown promising effects in terms of performance, but their effects on welfare have had little evaluation. In the present study, we aimed at evaluating the effect of diet supplementation on welfare indicators. A total of 246 piglets were weaned and transported for 12 h. After transport, they were assigned to one of 3 diets for a 14-day period: A—an antibiotic diet including chlortetracycline and tiamulin, NA—a control diet without any antibiotic or feed supplement, GLN—a diet including 0.20% L-glutamine. After the 14-day period, all piglets were fed the same diet. Tear staining was measured 11 times post-weaning (from d0 to 147). Skin lesions were counted before and after weaning (d-2, 2, and 36). Novel object tests (NOT) were done in groups 4 times post-weaning (d17, 47, 85, 111). Samples for 16S rRNA gene composition were collected prior to transport (d0), following the 14-day period (d14) and at the conclusion of the nursery phase (d34). The NA pigs appeared less interested in novel objects. On d17, they avoided the object less than A pigs (*P* < 0.05). They spent less time exploring the object on d85 and took longer to interact with the object on d111 than A and GLN pigs (*P* < 0.05). NA pigs also appeared more sensitive to environment and management. They had larger tear stains than GLN pigs on d84 and 110 (*P* < 0.05). On d2, NA pigs had more lesions than A and GLN (*P* < 0.01). In terms of microbiota composition, GLN had higher α-diversity than A and NA (*P* < 0.001). Differences between dietary treatments were absent at d0, were demonstrated at d14 and disappeared at d34. Pearson correlations between aggression, stress and anxiety indicators and bacterial populations were medium to high from 0.31 to 0.69. The results demonstrate that short-term feeding strategy can have both short- and long-term effects on behavior and welfare, that may partly be explained by changes in gut microbiota composition. Supplementation with GLN appears to confer similar benefits to dietary antibiotics and thus could be a viable alternative.

## Introduction

The routine use of antimicrobials in animal production has been identified as a potential factor in the development of antimicrobial resistance (1) and societal concern is driving increased stewardship, using the 5Rs model of responsibility, reduction, refinement, replacement, and review (2), especially in the use of medically-important antimicrobials. In the United States, increased stewardship has led to a 33% reduction in the domestic sale and distribution of all medically-important antimicrobials intended for use in food-producing animals, between 2016 and 2017 (3). However, certain swine production practices continue to impose increased risk of stress-induced disease susceptibility, and hence the use of antimicrobials is perceived to confer production advantages (4), although removal (5) or replacement with an alternative (6) may not be detrimental.

Stress can be defined as “an environmental effect on an individual which overtaxes its control systems and results in adverse consequences, eventually reduced fitness” (7). One of the most stressful events for pigs during production is that of weaning (8). At this time, they are subject to removal from the sow, an abrupt change in diet from milk to solid food and mixing into groups with unfamiliar pen-mates. In some countries, they may also be subjected to an additional stressor of transportation (9). These combined stressors often result in post-weaning diarrhea and compromised welfare post-weaning (10). The implication of various bacteria in post-weaning diarrhea has resulted in routine use of antimicrobials at this time-point, which impacts intestinal microbiota (11) but is seen to improve welfare, at least in the short term. The longer-term impact may be opposite. Reduced microbiome diversity and richness can reduce adaptability should the animal be challenged by dietary disturbances (12) or pathogens (13). This study used a combination of chlortetracycline, a medically-important antimicrobial aimed at prevention and treatment of gastrointestinal diseases, and tiamulin, a non-medically important antimicrobial aimed at prevention and treatment of respiratory diseases. This combination is the most commonly-prescribed antimicrobial mix used for 14d in post-weaning diets in the U.S., and has no adverse effects used at the concentrations prescribed. Alternatives to antimicrobials are being sought that will have the same health and welfare benefits, without the implications for antimicrobial resistance. These include phages, bacteriocins, peptides, probiotics, synthetic compounds, and nutraceuticals (14, 15). One potential nutraceutical is L-Glutamine, a conditionally-essential amino acid and immunomodulator that inhibits pro-inflammatory cytokines (16, 17). Dietary L-Glutamine supplementation can improve overall health and growth of newly weaned pigs (16–20) through a variety of mechanisms including: increased intestinal health and immune function, enhanced oxidative-defense capacity, prevention of intestinal atrophy, improved antibacterial activity, and greater nutrient absorption. It is well-known that L-Glutamine supplementation can improve piglet health and increase productivity (16–20), but little has been reported regarding its effects on swine welfare. In a study on simulated transport, weaned piglets fed diet with added L-Glutamine showed decreased intestinal damage, increased feed intake and growth, and decreased behaviors associated with illness compared to piglets provided diet without antibiotics (6). However, effects on other behaviors, and indicators of welfare and emotional state are not known.

Whereas, the potential effects of diet on behavior in pigs has been described (21), it is only in the last decade that the link between the gastrointestinal microbial population and brain function has begun to be elucidated (22, 23). We now know that the microbiome and the brain communicate bi-directionally *via* multiple suggested mechanisms, known collectively as the microbiome-gut-brain axis (24, 25). The existence of microbiome-gut-brain axis, and its relationship with behavior, affective state, cognition and stress response, is reported for numerous animal species, primarily rodents and humans. From human and other animal literature, the microbiome has been shown to play a role in anxiety and depression (26), fearfulness (27) and aggression (28) with shifts in microbiome linked to changes in these behaviors or affective states. As a route by which health and welfare of pigs can be manipulated, it is only recently receiving attention and is so far poorly understood (29, 30) but, theoretically, modification of the pig's gut microbiota offers a potential method to improve the pig's response to weaning and other lifetime stressors, and to improve overall health and welfare.

Since we know of the links between gut microbiome and fearfulness/anxiety and aggression, measures of interest will be behavioral responses during novel object tests carried out in the home pen so as to remove the confound of novel environment (31) and skin lesions post-mixing —a validated proxy measure of aggression (32). Tear staining will also be recorded, as the secretion of the Hardarian gland has been demonstrated as a potential tool for assessment of welfare in pigs on farm (33) and in a laboratory setting (34).

The objectives of the study were (1) To evaluate whether the administered diet can impact behavioral and welfare indicators during the administration period; (2) To determine if any diet effects can still be observed once the administration period is over; (3) To correlate behavioral indicators with specific microbiota composition.

## Materials and Methods

### Ethics Statement

All procedures involving animal use were approved by the Purdue University Animal Care and Use Committee (protocol #1603001385), and animal care and use standards were based upon the *Guide for the Care and Use of Agricultural Animals in Research and Teaching* (35).

### Animals and Management

A total of 246 (138 barrows, 111 gilts) crossbred Duroc × (Landrace × Yorkshire) piglets from 32 L, free from any visible health issues, were used in the study. At weaning (means ± SD: age 18 ± 4.2 d; weight 3.2 kg < 5.4 ± 1.4 kg < 8.9 kg), piglets were removed from the sow and herded up an 11.0° incline ramp into a gooseneck livestock trailer (2.35 × 7.32 m; Wilson Trailer Company, Sioux City, IA) at a density of 0.07 m^2/^pig. In the trailer, the ambient temperature was 11.0 ± 0.2°C and the relative humidity was 63.1 ± 0.9%. Trailers were bedded with wood shavings and ventilation openings were adjusted based on the ambient temperature (36). Piglets were transported as a group in the trailer for approximately 12 h and 819 km without feed or water. Total transport time was determined by adding loading time, time spent in the trailer, unloading time, and the time it takes to be sorted into their respective pens in the nursery facility. The procedure of transportation is described in Duttlinger et al. (37). Immediately following transport, 6 of them were euthanized and dissected (see the detailed procedure below) whereas the others weaned piglets were herded out of the trailer, weighed, and sorted into 30 pens of 8 pigs/pen (10 pens per treatment), blocked weight and balanced for sex. Pens (1.22 × 1.37 m) initially provided approximately 0.21 m^2^ per pig. All pens contain one 5-hole dry self-feeder and a cup waterer to allow for *ad libitum* access to feed and water. Each pen was assigned to 1 of the 3 diets fed in 2 phases for a 14-day period post-transport: A—an antibiotic diet including a common commercially prophylactic antibiotic treatment (n = 80 piglets; 441 ppm of chlortetracycline + 38.6 ppm tiamulin), NA—a diet without any prophylactic antibiotics or feed supplement (n = 80 piglets), GLN—diet including a nutraceutical diet containing L-glutamine (n = 80 piglets; 0.20% L-glutamine as-fed). Base diets were similar for all diet treatments and were formulated to meet nutritional requirements based on piglet BW with identical ME, CP, SID Lys, Ca and P across the 3 treatment diets (for further details see (37)). On d15, dietary treatments were ceased and all piglets were provided the same standard diet for the remainder of the study both in the nursery, fed in 2 phases, and in the grow-finish barn, fed in 6 phases [for further details see (37)]. During the first week after weaning, 1 NA pig died. During the second week, 1 pig from each treatment died. Another NA pig died 5 weeks post-weaning and a GLN pig was removed from the study at 7 weeks because of a lameness.

On d0, 6 piglets (3 males and 3 females) were euthanized. On d15, 30 piglets (1 piglet from each treatment pen) were euthanized, and an additional 30 pigs (1 piglet from each treatment pen) were euthanized on d34, at the end of the nursery period. Piglet selection for euthanasia was balanced for sex, weight and absence of medical treatment required during the nursery phase. Euthanasia was carried out by CO_2_ exposure using a Euthanex® AgPro™ (Euthanex Corp, Easton, PA, USA) followed by exsanguination. All euthanized piglets were dissected for gut contents and gut mucus collection. The remaining 6 pigs/pen (*n* = 180 pigs) were moved to a grow-finish building where they remained in the same 30 groups until market. The grow-finish facility contained pens (1.68 m × 4.27 m) that provided ~1.19 m^2^ per pig. All pens contained one 2-hole dry self-feeder and a nipple waterer to allow for *ad libitum* access to feed and water.

### Measurements

#### Tear Staining

Photographs of the left eye of each piglet were taken 11 times during the experiment to measure tear-staining: 24 to 48 h (d-2) before transport and on d2, 7, 15, 21, 28, 34, 47, 84, 110, and 146 after transport. Measurements were made on photographs by a single experienced person, blind to treatment, using the ImageJ software (38) to delimit the tear perimeter. The length of the iris was used as a scale to standardize the measurements. All the brownish areas on the direct periphery of the eye (bottom of the upper eyelid, top of the lower eyelid, internal and external corners) were recorded (34). The variable analyzed was the cumulative area covered by the stain.

#### Skin Lesions

Photographs of both sides of the body, as well as the head of each pig were taken 3 times during the experiment to measure skin body lesions: 24 to 48 h before transport (d-2) and on d2 and 39 after transport. Fresh skin lesions were counted on photographs by a single experienced person, blind to treatment, according to the Welfare Quality® Assessment Protocol in pigs (39) adapted for the first two stages as followed: all lesions above 1 cm were registered as 1 lesion; when at least 3 lesions below 1 cm were grouped within 2 cm, they were scored as 1 lesion. The variables analyzed were the total number of lesions in the front part of the body (as far back as the shoulder) and the cumulated number of skin lesions measured on the all body.

#### Novel Object Tests

Four 5-min novel object tests were carried out in the pigs' home pens. Four different objects, unfamiliar for the pigs, were used: a 20 cm swimming pool noodle, a 30 cm tall orange traffic cone, a 20 cm diameter PVC pipe wye socket and a 45 cm diameter aluminum trash can, respectively on d17, 47, 85, and 111 after transport. On the day prior to the test, all pigs were individually identified in each pen by a combination of 2 colors and 3 different locations (shoulder, back, hind-quarter) drawn on their body using livestock markers (All-Weather Paintstik™–La-Co Industries, Elk Grove Village, IL, USA). Immediately before introducing the object, the experimenter ensured all the pigs were standing and away from the feeder. However, some pigs did not see the object immediately when it was introduced in the pen. Therefore, variables regarding “interaction” with the object were corrected by the time needed for the pig to see the object for the first time (= head of the pig oriented toward the object with no contact with the object). The variables measured were: the number of withdrawals “Withdrawal” (sudden lateral or backward movements occurring with the head of the pig oriented toward the object), the latency to interact with the object for the first time “Interaction latency” (contact between the head of the pig and the object) which represents the interval between the first sight of the object and the first interaction with it, and the percentage of time spent interacting with the object “Interact duration” (total duration interacting with the object divided by the total duration from the first sight of the object to the end of the test). Video recordings were analyzed using the XP Observer 14 software (Noldus, The Netherlands) by a single experienced trained observer, blind to treatment.

#### Weighing Procedures

Once in the grow-finish barn, pigs were weighed every 3 weeks. Three of these handling procedures were videotaped on d55, 96, and 138. The gate of the pen was opened for 15 s with no human handling, and the number of pigs exiting the pen voluntarily was counted. After this period, a single experimenter, blind to treatment, entered the pen to bring out the remaining pigs and drive the group to the scale. The variables analyzed were: the number of pigs exiting the home pen voluntarily and the duration that it took the handler to remove all pigs from the pen ending with the rear feet of the last pig crossing the pen threshold “All out duration.” Video recordings were analyzed using the XP Observer 14 software (Noldus, The Netherlands) by a single experienced trained observer, blind to treatment.

#### Microbial Analyses of Gut Contents and Mucus

Samples for microbial analyses came from 6 piglets euthanized immediately following transport, 30 piglets (10 piglets per treatment) euthanized following the 14-d diet treatment period and from an additional 30 pigs (10 piglets per treatment) at the conclusion of the nursery phase (i.e., 20 d later).

We analyzed two kinds of samples for 16S rRNA gene composition: contents and mucus from three different locations in the gut (ileum, caecum and colon). The ileum part was a 10 cm section sampled 20 cm anterior to the ileal-cecal junction. The 10 cm colon section was collected on both sides of the top of the convolute of the ascending colon. The entire caecum was separated from the rest of the digestive tract. The tissue section was held on a board wrapped with autoclaved foil and cut through the longitudinal side. Gut contents samples were collected in sterile 2 ml tubes using sterilized spatulas, snap frozen with liquid nitrogen and stored at −80°C until DNA extraction. After collecting the gut content samples, the 10 cm intestinal tissue sections were washed with sterile PBS buffer until no remaining gut contents were visible. A sterile Cytosoft® cytology brush (Medical Packaging Corporation, Camarillo, CA, USA) was used to remove mucus and bacterial cells from the surface of the tissue. The brush was agitated in a 50 ml sterile Falcon tube containing 15 ml of PBS buffer and placed on ice. In the lab, the tubes were centrifuged at 5,000 g to pellet bacteria. The pellet was stored in 2 ml sterile tubes and frozen at −80°C until DNA extraction.

The DNA was extracted from 200 mg of each of the 6 frozen gut contents and mucus samples per pig by bead beating using the Fast DNA® SPIN kit for feces (MP Biomedicals Corporation, Irvine, CA, USA). Extracted DNA was then sent to the Argonne National Laboratory Environmental Sample Preparation and Sequencing Facility (Lemont, IL, USA) for PCR amplification of the V4 region of the 16S rRNA gene (515F-806R) (Forward: GTGYCAGCMGCCGCGGTAA; Reverse: GGACTACNVGGGTWTCTAAT) and sequenced using the MiSeq reagent kit V2 on an Illumina MiSeq (500 cycles) (Illumina Inc., San Diego, CA, USA). The sequencing library was generated using an integrated 12-base Golay barcode in the forward primer. Each 25 μL PCR reaction contains 9.5 μL of MO BIO PCR Water (Certified DNA-Free), 12.5 μL of QuantaBio's AccuStart II PCR ToughMix (2x concentration, 1x final), 1 μL Golay barcode tagged Forward Primer (5 μM concentration, 200 pM final), 1 μL Reverse Primer (5 μM concentration, 200 pM final), and 1 μL of template DNA. The conditions for PCR were as follows: 94°C for 3 min to denature the DNA, with 35 cycles at 94°C for 45 s, 50°C for 60 s, and 72°C for 90 s; with a final extension of 10 min at 72°C to ensure complete amplification.

#### Sequence Processing and Microbial Community Analyses

Briefly, 16S rRNA gene sequences were processed and clustered using the mothur v.1.39.3 standard operating procedure (SOP) designed for MiSeq data (40); the mothur MiSeq SOP was accessed in August 2018. The SILVA-based bacterial reference alignment (version 132) was used to identify the taxonomy of OTUs at a cluster cutoff of 97% sequence identity. Measures of richness, evenness and diversity were determined in mothur. Richness can be defined as the number of OTUs per sample, evenness represents the uniformity of the distribution of an individual amongst a community over different species, while α-diversity is a concept combining both richness and evenness (41). Richness and α-diversity were determined through the coverage, the number of OTUs observed, the Chao1, the ACE, the Shannon, the Simpson, the inverse Simpson estimators and β-diversity were calculated using the thetaYC distances as implemented in mothur. The OTU table was rarefied to 2,332 sequences per sample. A linear model with the treatment (A, GLN, NA), the day of sampling (14 or 34), the location (caecum, colon, ileum) and the type of sample (gut, mucus) was used for the richness, evenness and diversity indicator analyses. The effects of dietary treatments and days on the microbial community structure were tested using an analysis of molecular variance (AMOVA) of the thetaYC distance matrix in mothur. Results were adjusted by using the Bonferroni correction. The “metastats” command in mothur was then used to determine the OTUs responsible for the significant differences observed using AMOVA. The “corr.axes” and “otu.association” commands in mothur, specifying the default Pearson method, were then used in combination to estimate the significant Pearson correlations between behavioral indicators and bacterial populations.

### Statistical Analysis

Statistical analyses were performed with the software R 3.4.3 (42). The variables of area of tear staining and the latency to interact with the novel object were normalized by logarithmic transformation before statistical analysis. Other variables were normal without transformation. In all the statistical analysis, *P* < 0.05 was considered statistically significant and 0.05 ≤ *P* < 0.1 as a trend.

Mixed effects model for repeated measures with the treatment, the day of sampling and the interaction between the two factors as fixed effects and the pen and the animal being included as random effects were used with the tear staining area, skin lesions and novel object test variables. The statistical unit for the previous traits was the animal. Regarding the weighing procedures variables, a second mixed effects model for repeated measures was used with the treatment, the day of sampling, the number of pigs per pen (5 or 6) and the interaction between the treatment and the day as fixed effects and the pen being included as random effects. The statistical unit for the weighing procedures variables was the pen. These analyses were done with the function lmer from the R package “lme4” 1.1-7. The emmeans function from the R package “emmeans” 1.2-2 was used to perform pairwise comparisons with the FDR correction when interactions were significant (*P* < 0.05) or were tendencies (*P* < 0.1).

## Results

Ages and weights at different days are given as indications of performance in Table 1. Means, SEM, minimum and maximum of all untransformed data are reported in Table 1.

**Table 1:** Numbers (N), means, standard errors of the means (SEM), minimum (Min), maximum (Max) of welfare indicators or behaviors measured during tests, all dietary treatments mixed.

**Family of traits**	**Traits**	***N***	**Day**	**Mean ± SEM**	**Min**	**Max**
Tear staining	Area (cm^2^)	239	d-2	0.067 ± 0.003	0	0.3
		238	d2	0.063 ± 0.004	0	0.5
		238	d7	0.061 ± 0.004	0	0.4
		206	d15	0.072 ± 0.005	0	0.5
		206	d21	0.081 ± 0.005	0.007	0.4
		205	d28	0.13 ± 0.01	0.008	2.7
		171	d34	0.17 ± 0.01	0.007	0.7
		171	d47	0.40 ± 0.03	0	2.8
		174	d84	0.66 ± 0.04	0.009	3.0
		174	d110	0.73 ± 0.04	0.02	2.6
		170	d146	0.85 ± 0.06	0.01	6.5
Lesions	Front (N)	240	d-2	0.85 ± 0.20	0	30
		240	d2	21.01 ± 0.70	0	65
		174	d39	0.78 ± 0.20	0	10
	Total (N)	240	d-2	0.91 ± 0.20	0	31
		240	d2	21.74 ± 0.80	0	73
		174	d39	1.48 ± 0.20	0	15
Novel object test	Withdrawal (N)	201 160	d17 d47	1.34 ± 0.20 0.58 ± 0.10	0 0	18 11
		174	d85	0.27 ± 0.08	0	13
		175	d111	0.95 ± 0.10	0	9
	Interaction duration (%)	201 162	d17 d47	83.40 ± 0.01 82.60 ± 0.01	4.4 2.4	100 100
		174	d85	72.10 ± 0.01	8.4	100
		175	d111	78.60 ± 0.01	9.5	100
	Interaction latency (s)	188 155	d17 d47	10.3 ± 1.7 4.20 ± 0.45	0.087 0.44	175 55
		169	d85	2.60 ± 0.28	0.23	21
		172	d111	6.4 ± 1.0	0.30	137
Weighing	Pig voluntary out (N)	30 30	d55 d96	3.2 ± 0.3 3.3 ± 0.3	0 0	5 6
		30	d138	3.3 ± 0.2	1	6
	All out duration (s)	30 27	d55 d96	10.1 ± 1.0 7.4 ± 1.0	0.5 0.8	22.2 18.6
		28	d138	11.5 ± 1.1	1.6	24.8

### Tear Staining

The day of sampling was significant (*P* < 0.001) with higher values as the pigs aged (Table 2). On d84, tear staining areas of the NA pigs were larger than GLN pigs (*P* = 0.022) and tended to be higher than A pigs (*P* = 0.070). On d110, NA pigs had larger tear staining areas than GLN pigs (*P* = 0.003) and tended to have larger tear staining areas than A pigs (*P* = 0.080). No other significant effects were found (*P* > 0.1).

**Table 2:** Effect of the diets (A: an antibiotic diet including 441 ppm of chlortetracycline and 38.6 ppm tiamulin; NA: a control diet without any prophylactic antibiotic or feed supplement; GLN: a diet including 0.20% L-glutamine as-fed) and the day (from 2 d before transport of weaned piglets to 147 d after) for repeated measures on tear staining area, numbers of skin lesions, and novel object test variables (155 < *n* < 240)^a^.

**Test**	**Traits^**b**^**	**Diet**	**Day**	**Diet × Day**	**Effects**^****c****^	
Tear staining	Log (Area) (cm^2^)	NS	***	NS	d-2, 2, 7, 15, 21, 28, 34, 47, 84, 110, 146	NS
Lesions	Front (N)	NS	***	**	d-2	NS
					d2	NA > GLN^***^, A^*^ NA = 4.8 ± 0.6 A = 3.1 ± 0.4 GLN = 2.5 ± 0.4
					d36	NS
	Total (N)	NS	***	***	d-2	NS
					d2	NA > GLN^***^, A^**^ NA = 25.3 ± 1.4 A = 21.1 ± 1.4 GLN = 18.8 ± 1.3
					d36	NS
Novel object test	Withdrawal (N)	*	^#^	^#^	d17	NA < GLN^#^, A^*^ NA = 0.84 ± 0.2 A = 1.64 ± 0.3 GLN = 1.58 ± 0.4
					d47, 85, 111	NS
	Interaction duration (%)	NS	***	**	d17, 47	NS
					d85	NA < GLN*, A^**^ NA = 12.3 ± 2.4 A = 18.9 ± 4.1 GLN = 13.5 ± 3.3
					d111	NS
	Log (Interaction latency) (s)	NS	***	^*^	d17, 47, 85	NS
					d111	NA > GLN^**^, A^*^ NA = 15.7 ± 3.0 A = 11.3 ± 3.1 GLN = 8.5 ± 1.2

a Statistical linear repeated model formula: Trait ~ Diet + Day + Diet × Day + Random(Pen) + Random(Pig). NS: P > 0.1;

# : P < 0.1;

* : P < 0.05;

** : P < 0.01;

*** *: P < 0.001. Pig is the statistical unit*.

b *Traits: Log (Area) = log(area of the tear staining + 1); Log(Interaction latency) = log(latency to interact with the object)*.

c *Adjusted means ± SEM*.

### Skin Lesions

The interaction between the diet and the day of sampling was significant both for the front (*P* = 0.002) and the total (*P* < 0.001) lesions numbers (Table 2). No significant effect was found on d-2 and d39 (*P* > 0.1). On d2, NA piglets had more front skin lesions than GLN (*P* < 0.001) and A piglets (*P* = 0.011), as well as more total number of skin lesions than GLN (*P* < 0.001) and A piglets (*P* = 0.005).

### Novel Object Tests

The interaction between the diet and the day of sampling was significant for the duration of interaction (*P* = 0.004) and the latency to interact with the object for the first time (*P* = 0.013) and there was a tendency for the number of withdrawal behaviors (*P* = 0.055) (Table 2). On d17, NA piglets avoided the object less than A piglets (*P* = 0.033) and tended to avoid it less than GLN piglets (*P* = 0.058). No significant effect was found on d47. On d85, NA pigs spent less time interacting with the object than GLN (*P* = 0.025) and A pigs (*P* = 0.008). On d111, NA pigs took a longer time to interact for the first time with the object than GLN (*P* = 0.002) and A pigs (*P* = 0.037).

### Weighing Procedures

The interaction between the diet and the day of sampling was not significant, as well as the treatment and the day of sampling regarding both the number of pigs voluntarily exiting the pen or the duration needed for the handler to push out all the pigs from the pen (*P* > 0.1) (data not shown).

### Microbiota Analyses

#### Richness, Diversity, and Composition of the Gut Microbiota

The richness, evenness and diversity indexes of the gut bacterial community varied with dietary treatment (A, NA, GLN), time (d0, d14 or d34), location (caecum, colon, ileum) and type (lumen content, mucus) (Table 3). GLN pigs had higher richness, evenness and diversity than A and NA pigs (*P* < 0.001). Richness, evenness and diversity changed over time (*P* < 0.001). Across both luminal and mucosal samples, the colon had the highest richness, evenness and diversity (*P* < 0.001). Mucosal samples were richer, more even and more diverse than gut content samples (*P* < 0.001). The average number of OTUs per sample was 150 ± 4. Good's coverage estimate was 97.5 ± 0.06% across all samples.

**Table 3:** α-diversity indicators of the microbiota in gut content and mucosal samples, at three different locations (Caecum, Colon, Ileum), at two different days (d14 and d34) after the beginning of three different two-week diets (A: an antibiotic diet including 441 ppm of chlortetracycline and 38.6 ppm tiamulin; NA: a control diet without any prophylactic antibiotic or feed supplement; GLN: a diet including 0.20% L-glutamine as-fed)^a^.

**Traits**	**Treatment**^**b**^				**Day**^**b**^				**Location**^**b**^				**Type**^**b**^		
	**A**	**GLN**	**NA**	***P***	**d0**	**d14**	**d34**	***P***	**Caecum**	**Colon**	**Ileum**	***P***	**Gut content**	**Mucus**	***P***
Chao1 estimator	230.7 ± 8.3**a**	267.5 ± 8.2**b**	230.7 ± 5.9**a**	***	263.7 ± 12.7**b**	222.6 ± 5.5**a**	242.6 ± 5.7**b**	**	262.7 ± 7.1**b**	317.2 ± 7.4**c**	149.0 ± 74**a**	***	226.5 ± 6.4**a**	259.4 ± 6.1**b**	***
ACE estimator	264.4 ± 11.0**a**	317.9 ± 10.9**b**	273.4 ± 7.8**a**	***	300.1 ± 17.0	267.9 ± 7.4	287.8 ± 7.6	NS	290.6 ± 9.5**b**	363.5 ± 9.8**c**	201.6 ± 9.9**a**	***	266.7 ± 8.6**a**	303.8 ± 8.2**b**	***
Shannon index	3.25 ± 0.06**a**	3.42 ± 0.06**b**	3.26 ± 0.04**a**	*	3.48 ± 0.09**b**	3.27 ± 0.04	3.19 ± 0.04**a**	**	3.74 ± 0.05**b**	3.97 ± 0.05**c**	2.23 ± 0.05**a**	***	3.17 ± 0.04**a**	3.46 ± 0.04**b**	***
Simpson index	0.122 ± 0.01	0.103 ± 0.009	0.117 ± 0.007	NS	0.105 ± 0.01**ab**	0.102 ± 0.006**a**	0.136 ± 0.007**b**	***	0.055 ± 0.008**a**	0.046 ± 0.008**a**	0.241 ± 0.008**b**	***	0.129 ± 0.007**b**	0.100 ± 0.007**a**	***
InvSimpson index	16.4 ± 0.9**a**	19.8 ± 0.8**b**	16.3 ± 0.6**a**	***	19.8 ± 1.3**b**	16.3 ± 0.6**a**	16.4 ± 0.6**a**	*	20.5 ± 0.7**b**	25.1 ± 0.8**c**	6.8 ± 0.8**a**	***	16.2 ± 0.7**a**	18.8 ± 0.6**b**	***
Number of OTUs	148.4 ± 5.0**a**	169.8 ± 4.9**b**	148.3 ± 3.6**a**	***	168.9 ± 7.7**b**	140.9 ± 3.3**a**	156.8 ± 3.4**b**	***	173.0 ± 4.3**b**	207.4 ± 4.5**c**	86.2 ± 4.5**a**	***	143.2 ± 3.9**a**	167.8 ± 3.7**b**	***
Coverage (%)	97.55 ± 0.1**b**	97.12 ± 0.1**a**	97.53 ± 0.07**b**	***	97.26 ± 0.1**ab**	97.63 ± 0.06**b**	97.32 ± 0.07**a**	**	97.25 ± 0.08**b**	96.57 ± 0.09**a**	98.39 ± 0.09**c**	***	97.61 ± 0.07**b**	97.20 ± 0.07**a**	***

a Statistical linear model formula: Trait ~ Treatment + Time + Location + Type. Letters were attributed for significantly different values a < b < c. NS: P > 0.1;

* P < 0.05;

** P < 0.01;

*** *P < 0.001*.

b *Adjusted means ± SEM*.

Three different phyla were found across all samples: *Firmicutes* represented 77.5% of the total 16S rRNA gene sequences, *Bacteroidetes* 13.5% and *Proteobacteria* 4.9%. All the remaining phyla represented <2%. The major classes of the *Firmicutes* phylum were *Clostridia* (57.6% of the sequences), *Bacilli* (28.6%), *Negativicutes* (9.4%) and *Erysipelotrichia* (3.7%); of the *Bacteroidetes* phylum were *Bacteroidia* (95.4%) and *Bacteroidetes* unclassified (4.6%); of the *Proteobacteria* phylum were *Gammaproteobacteria* (49.7%), *Proteobacteria* unclassified (23.0%), *Epsilonproteobacteria* (19.7%), *Betaproteobacteria* (3.6%), and *Deltaproteobacteria* (3.4%).

#### Effects of Treatments, Time, Location, Type on Microbiota

**Figure 1:**
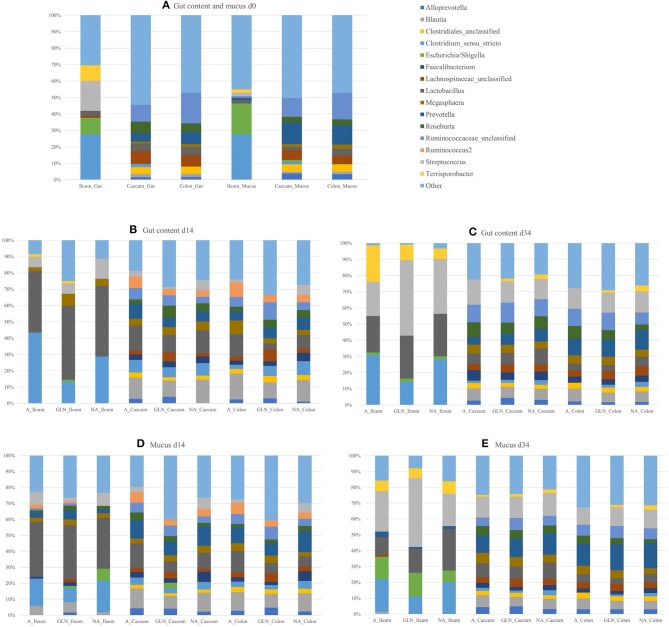
Relative abundances of genera for the three dietary treatments (A: an antibiotic diet including 441 ppm of chlortetracycline and 38.6 ppm tiamulin; NA: a control diet without any prophylactic antibiotic or feed supplement; GLN: a diet including 0.20% L-glutamine as-fed), plotted by day of sampling (d0, d14, and d34), location of samples (Caecum, Colon, Ileum) and type of samples (gut content and mucus): **(A)** d0, **(B)** Gut d14, **(C)** Gut d34, **(D)** Mucus d14, and **(E)** Mucus d34. Genera outside the 15 most abundant are combined as “Other”.

**Figure 2:**
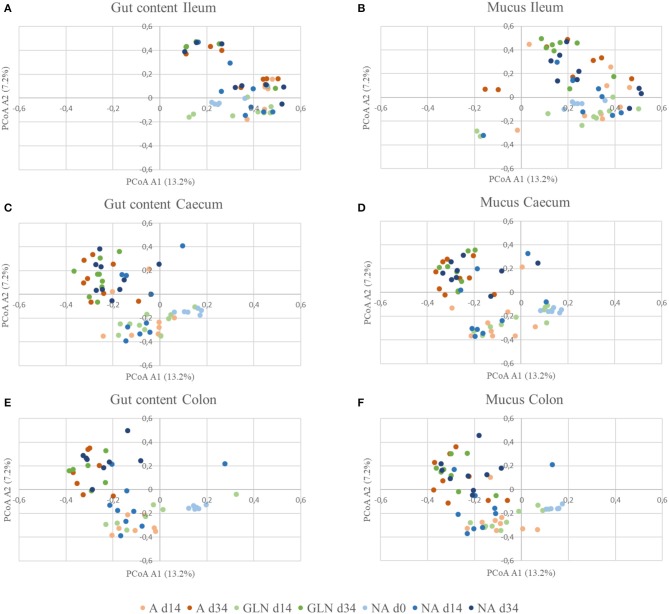
β-diversity of gut communities with respect to diet, location, and sample type. Distances were calculated using the Yue and Clayton theta metric and plotted using PCoA for the three dietary treatments (A: an antibiotic diet including 441 ppm of chlortetracycline and 38.6 ppm tiamulin; NA: a control diet without any prophylactic antibiotic or feed supplement; GLN: a diet including 0.20% L-glutamine as-fed) on three day of sampling (d0, d14 and d34) plotted by location and type of samples: **(A)** Gut Ileum, **(B)** Mucus Ileum, **(C)** Gut Caecum, **(D)** Mucus Caecum, **(E)** Gut Colon and **(F)** Mucus Ileum.

**Figure 3:**
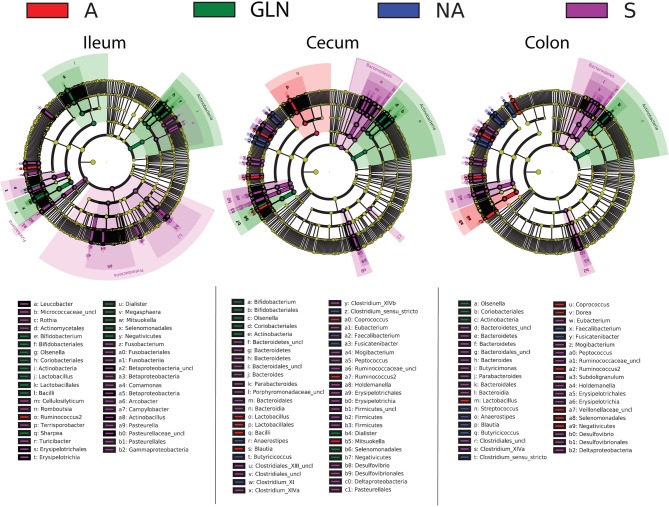
Linear discriminant analysis of taxa differentiating the pigs prior to dietary treatment (S: Sentinel pigs) and of the three dietary treatments at d14 (A: an antibiotic diet including 441 ppm of chlortetracycline and 38.6 ppm tiamulin; NA: a control diet without any prophylactic antibiotic or feed supplement; GLN: a diet including 0.20% L-glutamine as-fed) by gut location (both lumen and mucus combined). Taxa with LDA scores > 3.5 as computed via LEfSe are plotted on the cladograms. Unclassified taxa are referenced as “uncl”.

The microbial community structure is graphically presented in Figures 1–3, respectively with a stacked bar chart, PCoA plots and a taxonomic LEfSe. The effects of dietary treatments, day of sampling, location of samples, type of samples and the interaction between dietary treatment and day on gut microbiota composition are presented in Table 4. Days 0, 14, and 34 varied in terms of microbiota composition in the caecum and colon for both lumen content and mucus samples (*P* < 0.05). For both lumen content and ileal mucosa samples, the microbiota composition was stable between d14 and 34 time, except for the GLN treatment. Within a day and a type of sample, ileum samples differed from caecum and colon samples (*P* < 0.001). Differences between dietary treatments were demonstrated on d14 but had disappeared by d34. GLN-fed piglets had higher percentages of *Actinobacteria* and *Firmicutes* (*P* < 0.001) but there were no differences in terms of percentages of *Bacteroidetes* and *Proteobacteria* (*P* > 0.1).

**Table 4:** Effects of dietary treatments (A: an antibiotic diet including 441 ppm of chlortetracycline and 38.6 ppm tiamulin; NA: a control diet without any prophylactic antibiotic or feed supplement; GLN: a diet including 0.20% L-glutamine as-fed), day of sampling (d0, d14, and d34), location of samples (Caecum, Colon, Ileum) and type of samples (gut content and mucus) on the gut microbiota^a^.

	**Type**	**Location**	**Treatment**	**Day^**b**^**	**Treatment × Day^**b**^**	**Bacterial taxa differences for significant treatment** **×** **Day interactions (*****Genus level*****)**^****c****^	
1	Gut content	Caecum	NS	d0 ≠ d14 ≠ d34 ^**^	A_ d14 ≠ GLN_ d14 ^*^	*Kitasatospora*	A>GLN
						*Lachnospiraceae unclassified*	A>GLN
						*Lactobacillus*	A<GLN
						*Veillonellaceae unclassified*	A<GLN
2	Mucus	Caecum	NS	d0 ≠ d14 ≠ d34 ^***^	A_ d14 ≠ GLN_ d14 ^*^	*Lachnospiraceae unclassified*	A>GLN
						*Paraprevotella*	A<GLN
						*Ruminococcaceae unclassified*	A<GLN
						*Treponema*	A<GLN
3	Gut content	Colon	NS	d0 ≠ d14 ≠ d34 ^**^	A_ d14 ≠ GLN_ d14 ^*^		
4					A_ d14 ≠ NA_ d14 #		
5	Mucus	Colon	NS	d0 ≠ d14 ≠ d34 ^***^	A_ d14 ≠ GLN_ d14 ^*^	*Paraprevotella*	A<GLN
						*Ruminococcaceae unclassified*	A>GLN
6					A_ d14 ≠ NA_ d14 ^*^	*Alloprevotella*	A>NA
						*Bacteroides*	A>NA
						*Butyricicoccus*	A<NA
						*Clostridiales unclassified*	A<NA
						*Firmicutes unclassified*	A<NA
						*Ruminococcaceae unclassified*	A<NA
						*Treponema*	A<NA
7					GLN_ d14 ≠ NA_ d14 ^*^	*Butyricicoccus*	GLN<NA
						*Erysipelotrichaceae unclassified*	GLN>NA
						*Paraprevotella*	GLN>NA
8	Gut content	Ileum	NS	d0 ≠ d14 and d34 ^***^	GLN_d14 ≠ GLN_d34 ^***^	*Bifidobacterium*	d14>d34
						*Coriobacteriaceae unclassified*	d14>d34
						*Lactobacillus*	d14>d34
						*Streptococcus*	d14<d34
9	Mucus	Ileum	NS	NS	A_d14 ≠ A_d34 ^*^	*Bifidobacterium*	d14>d34
						*Helicobacter*	d14<d34
						*Lachnospiraceae unclassified*	d14<d34
						*Lactobacillus*	d14>d34
						*Prevotellaceae unclassified*	d14<d34
						*Ruminococcus2*	d14>d34
						*Tepidimonas*	d14>d34
						*Veillonella*	d14>d34
10					GLN_d14 ≠ GLN_d34 ^*^	*Bacteroidetes unclassified*	d14>d34
						*Bifidobacterium*	d14>d34
						*Clostridium sensu stricto*	d14>d34
						*Dorea*	d14>d34
						*Lactobacillus*	d14>d34
						*Prevotella*	d14>d34
						*Ruminococcus2*	d14>d34
						*Streptococcus*	d14<d34
						*Veillonella*	d14>d34
						*Veillonellaceae unclassified*	d14>d34
11					GLN_d34 ≠ NA_d34 #	*Bacteroidetes unclassified*	GLN<NA

a *Analysis of molecular variance (AMOVA) using the standardized distance matrix method in mothur adjusted by using Bonferroni correction. The determination of the bacteria responsible for the AMOVA significant differences were analyzed with the command “metastats” in mothur from the mothur standard operating procedure (SOP) designed for MiSeq data (40). The mothur MiSeq SOP was accessed in August 2018*.

b NS: P > 0.1;

# P < 0.1;

* P < 0.05;

** P < 0.01;

*** *P < 0.001*.

c *Bacterial taxa mentioned had a P < 0.001*.

#### Relation Between Bacterial Taxa and Behavioral Traits

Pearson correlations between aggression, stress and anxiety indicators and bacterial taxa were medium to high from 0.31 to 0.69 (Table 5). The number of total lesions 2 days before the start of the dietary treatment was correlated to the bacterial family *Acidaminococcaceae* (*r* = 0.65). Two days after the start of the dietary treatment, the number of total lesions was correlated with the *Prevotellaceae* family (*r* = 0.40) and the tear staining area was correlated with the order *Clostridiales* (*r* = 0.35). Significant correlations were found with variables up to 28 d after weaning. On d16, the number of withdrawals during the NOT was correlated with the *Lachnospiraceae* family (*r* = 0.69). The tear staining area was correlated with the family *Porphyromonadaceae* on d21 (*r* = 0.52) and with *Acidaminococcaceae* on d28 (*r* = 0.35).

**Table 5:** Significant Pearson correlations between welfare and behavioral traits and bacterial taxa^a^.

**Traits**	***p*-value^**b**^**	**Relative abundance (%)**	***R***	**Classified OTUs statistically associated with behavioral traits *(Genus level)*^**c**^**
Total lesions 2 days before treatment (N)	^**^	0.01	0.43	*Clostridium_IV*
	^**^	0.06	0.43	*Coriobacteriaceae unclassified*
	^**^	<0.01	0.42	*Fibrobacter*
	^**^	2.62	0.47	*Lachnospiraceae unclassified*
	^**^	12.05	0.43	*Lactobacillus*
	^**^	3.87	0.43	*Megasphaera*
	^**^	0.83	0.43	*Porphyromonadaceae unclassified*
	^**^	7.87	0.43	*Prevotella*
	^**^	0.26	0.63	*Ruminococcaceae unclassified*
	^**^	9.84	0.43	*Streptococcus*
	^**^	<0.01	0.65	*Succiniclasticum*
	^**^	<0.01	0.37	*Terrisporobacter*
Total lesions on d2 (N)	^*^	7.87	0.40	*Prevotella*
	^*^	0.29	0.31	*Ruminococcus*
Withdrawal during the Novel Object test on d16 (N)	^**^	<0.01	0.36	*Actinobacteria unclassified*
	^**^	0.04	0.32	*Alistipes*
	^**^	0.02	0.33	*Allisonella*
	^**^	2.20	0.57	*Alloprevotella*
	^**^	0.34	0.31	*Anaerostipes*
	^**^	0.43	0.36	*Bacteroides*
	^**^	6.21	0.35	*Blautia*
	^**^	<0.01	0.36	*Burkholderiales unclassified*
	^**^	1.73	0.36	*Butyricicoccus*
	^**^	<0.01	0.36	*Clostridium_XlVa*
	^**^	0.10	0.36	*Clostridium_XlVb*
	^**^	0.92	0.33	*Dorea*
	^**^	0.10	0.40	*Erysipelotrichaceae unclassified*
	^**^	2.58	0.36	*Faecalibacterium*
	^**^	0.01	0.36	*Faecalicoccus*
	^**^	2.62	0.69	*Lachnospiraceae unclassified*
	^**^	12.05	0.51	*Lactobacillus*
	^**^	3.87	0.36	*Megasphaera*
	^**^	0.55	0.36	*Phascolarctobacterium*
	^**^	7.87	0.36	*Prevotella*
	^**^	3.73	0.36	*Roseburia*
	^**^	0.29	0.35	*Ruminococcus*
	^**^	<0.01	0.54	*Ruminococcaceae unclassified*
	^**^	9.84	0.36	*Streptococcus*
	^**^	<0.01	0.36	*Subdoligranulum*
Tear staining area on d2	^*^	0.17	0.31	*Alloprevotella*
	^*^	1.95	0.35	*Clostridiales unclassified*
Tear staining area on d21	^**^	<0.01	0.49	*Clostridium_XlVa*
	^**^	2.62	0.50	*Lachnospiraceae unclassified*
	^**^	0.15	0.31	*Oscillibacter*
	^**^	0.02	0.52	*Porphyromonadaceae unclassified*
	^**^	7.87	0.37	*Prevotella*
	^**^	0.29	0.47	*Ruminococcus*
	^**^	0.34	0.41	*Treponema*
	^**^	3.87	0.31	*Megasphaera*
	^**^	0.26	0.33	*Ruminococcaceae unclassified*
	^**^	<0.01	0.35	*Succiniclasticum*

a *Significant OTU associations with behavioral traits were analyzed using the commands “otu.association” in mothur from the mothur standard operating procedure (SOP) designed for MiSeq data (40). The mothur MiSeq SOP was accessed in August 2018*.

b #P < 0.1;

* P < 0.05;

** P < 0.01; ***P < 0.001.

c *Bacteria mentioned had a P < 0.001*.

## Discussion

Specific feeding strategies for pigs to improve their ability to cope with stress or to alter behavior is a relatively unexplored area. In most studies dealing with feeding and stress and behavior in pigs, dietary changes have aimed to affect the time spent eating and the feeling of satiety to reduce aggressions, stress (43) and aberrant behaviors (44). However, there are a few studies that have emerged over the past 20 years that have revealed that dietary components *per se* can influence behavior.

Aggressiveness can be modulated *via* mineral intake, such as magnesium (45–47) and tryptophan supplementation (48–51). Besides preventing agonistic interactions from happening, an optimal ratio of fat, cholesterol and carbohydrate can even promote positive non-agonistic social interactions (52). Stress levels and fearful emotions can also be decreased with a large range of feed supplementation: vitamin E (46, 53), magnesium (47), tryptophan (48, 54–56), aromatic plant extracts (53, 57), chitosan (58), and the ratios of fat, cholesterol, carbohydrate (52), and linoleic acid in the diet (59). Finally, tryptophan has also demonstrated consistent effects in terms of reduction of aberrant behaviors, such as tail biting (49, 60), as well as changes in exploration in behavioral tests (61, 62). Exploration has also been increased by high linoleic acid ratio (59) or dietary cholesterol supplementation (63, 64). In the present study, pigs received three different diets: a diet including supplementary L-glutamine (GLN), a diet including an antibiotic treatment composed of chlortetracycline and tiamulin (A), and a diet without any prophylactic antibiotics or feed supplements (NA). The antibiotics and the L-glutamine were provided in feed. To the best of our knowledge, only one study recorded the behavior (excluding feeding or drinking) of pigs raised with either no supplementation in their feed, or orally supplemented with a common commercial prophylactic antibiotic or with L-glutamine (6). The authors demonstrated an increase in lying behavior and a decrease in standing behavior for NA pigs for the 48-h following a combined weaning and transport day. Those changes in behavior were correlated with reduced growth performance compared to GLN and A groups. They did not find any differences between the A and GLN groups. They suggested that these changes in behaviors for the NA group could reflect a higher degree of illness. Three welfare indicators—two of which are behavioral—have been recorded in the current study: tear staining, as a non-invasive indicator of stress (65); number of lesions, as an indirect indicator of aggressive behavior (32); and behavior during a novel object test, as an indicator or fear or anxiety (31). Similar to the study of Johnson and Lay (6), the NA treatment was the only treatment to differ from the other treatments. Oral antibiotics or L-glutamine provided similar beneficial effects regarding stress as minerals, amino-acids or plants extracts mentioned above. The NA piglets also had a higher number of lesions located in the front part of their body, as well as total lesions 48-h after mixing, representing more overall aggression in this treatment, with pigs likely more engaged in fighting (66). Results about aggression were only visible 48-h after mixing coinciding with the establishment of the hierarchy (67). Both A and GLN diets conferred similar positive effects in terms of aggression, as magnesium (45–47) and tryptophan supplementations (48–51). Finally, piglets supplemented with both A or GLN showed more withdrawals to a novel object dropped in their home pen at d16, but interacted longer with a novel object at d85 and interacted sooner with a novel object at d111. The three behaviors mentioned demonstrated a higher interest of the A and GLN treatment pigs for a novel object no matter their age and the time gap after the end of the feed supplementation. The higher number of withdrawals, in this particular situation, could also be interpreted as an increase in interest for the novel object as withdrawal appeared because of direct touching of the object that made it move. NA pigs adopted a strategy of flight in a corner of the pen. A better measure would have been the duration of cowering.

Feed supplements can be powerful tools as some studies have demonstrated effects on behavioral and welfare indicators only a few hours (62) or a few days after the beginning of the supplementation (46, 48–51, 54, 55). However, the majority of the studies have used a longer inclusion time of a few weeks (47, 52, 53, 56–61, 63, 64). Unfortunately, all the data collection is usually done only during the supplementation period or sometimes for a few days after. There is a critical knowledge gap about the longer-term effects of specific feed supplements over time. In the present study, the effects of short-term dietary treatments on stress and fear indicators were still significant up to 97 d after the end of the supplementation period. Inclusion of A or GLN treatments for 14 d induced similar short and long term effects on stress and behavioral indicators (tear staining, lesions and reactions during a novel object test). Those effects may come from changes in the microbiota composition. Indeed, GLN is a key regulator of microbiota composition as it participates in the nitrogen balance in the gut, which regulates the metabolism of bacteria (68). GLN promoted microbial richness and diversity in comparison to the A and NA treatments. Reduction of gut microbiota richness and diversity was associated with depression and anxiety-like behaviors in rodents (69, 70). In a study with obese humans, supplementation in 14 d with GLN induced a shift for the phyla *Actinobacteria* and *Firmicutes*, especially for the genera *Dialister, Dorea, Pseudobutyrivibrio*, and *Veillonella* (71). Shifts in the *Firmicutes* and *Bacteroidetes* phyla have also been observed in mice supplemented for 14 d with 1% L-glutamine (72). In the present study, pigs fed with the two dietary treatments A and GLN differed in terms of gut microbiota composition, while pigs from the NA group did not differ from the two other groups. *Firmicutes* was also the phylum mainly discriminating GLN and A groups after the 14-day dietary supplementation, followed by the phyla *Actinobacteri*a, *Bacteroidetes, Proteobacteria*, and *Spirochaetes*. Shifts in bacterial populations were visible across all gut locations, ileum, caecum and colon, and in both gut content samples and mucus samples. Shifts over time and along the entire gut tract, observed in our study, is consistently reported in swine microbiota studies (73). The microbial composition is following a dynamic process around weaning, mainly depending on changes from liquid to solid feed (74, 75).

Antibiotics are known to strongly affect the microbiota composition by the depopulation of sensitive bacterial (58, 76). This reduction in diversity and bacterial abundances of the gut microbiota disrupts the gut ecosystem and weakens host immunity to pathogens (77, 78). In the current study, A treatment pigs had a richness and diversity comparable to NA pigs and significantly lower than GLN pigs. The lower richness and diversity of the gut microbiota in NA pigs could indicate intestinal disruptions caused by a high pathogenic load. Overall, α-diversity reported in the present study, matched results demonstrated in comparable pig studies. There was an ascending gradient of α-diversity, richness and evenness from the upper part (ileum) to the lower part (caecum and colon) of the gut tract (79–85). In term of microbial composition, ileum samples were drastically different from caecum and colonic samples which were highly similar (81, 83, 84, 86, 87). Moreover, in the present study we also confirmed that mucosa samples have higher richness than lumen samples regardless of the location in the gut tract (82, 85, 88). The three most abundant phyla determined in this study: *Firmicutes, Bacteroidetes*, and *Proteobacteria* also matched what is always reported for pigs at that age (73, 79, 82, 86, 87, 89). In terms of behavioral traits, GLN and A groups behaved similarly and were different from NA pigs, while in terms of microbiota composition the two groups were contrasted. L-glutamine conferred similar effects to antibiotics in terms of welfare and behavior, and should therefore be considered as a serious alternative to antibiotics, which may cause a decrease in microbiota diversity, select for antibiotic resistance, as well as increase the risk of colonization by pathogenic bacteria especially because of the creation of free ecological niches (90).

On d34, i.e., 20 d after the end of the dietary treatment, the differentiation of microbiota composition between dietary groups observed on d14 was not noticeable anymore. It could derive from two main causes: a cross contamination between groups, due to the fact that pigs in the neighboring pens had limited physical contact, leading to a convergence in gut microbiota composition; or an inevitable reversion to the commensal microbiota composition because of a powerful gut microbiota homeostasis. The first hypothesis is possible in the present study as pigs were penned separately by dietary treatment groups but were all located in the same room, which made cross contamination possible. Despite this loss of difference in microbiota composition, behavioral effects were still visible up to 97 d after the end of the dietary treatment. One possible explanation is related to the effect of microbiota on epigenetics. Indeed, it has already been demonstrated that microbial metabolism of diet can affect host gene expression *via* epigenetics pathways (91), including genes involved in the regulation of locomotor activity and anxiety-like behaviors, such as time spent in light in a light/dark box test compartment or open arms in an elevated plus maze test (92). During the supplementation period, substrates available for methylation can, for example, be altered or the activity of enzymes can be modulated by the presence of unusual compounds. Those alterations will then modify persistently the gene expression, as it will be transferred from one cell division to the next one enabling long term heritable changes. To promote long term gut microbiota differentiation, supplementation should also preferentially be given during the perinatal period, when the vulnerability of the entire organism is enhanced (92).

The gut microbiota plays a crucial role in the regulation of brain development, as well as in the emotional state of individuals (92–95). Relationships have been demonstrated between pathological emotional behaviors, such as anxiety and depression, and gastrointestinal disorders like irritable bowel syndrome (23). Challenges of rodents with pathogenic bacteria have provoked changes in behaviors within a few hours, resulting in an increase in anxiety and a decrease in exploratory behaviors (96, 97) *via* a direct action on the vagus nerve (98–100). However, Bercik et al. (101) also showed an effect of microbiota composition on emotional behavior of mice with a sectioned vagus nerve, revealing the existence of other modes of communication between the microbiota and the HPA axis (23, 102). Several studies using the rodent model demonstrated the importance of the microbiota on the emotional state of individuals, especially anxiety (97, 101–106). In humans, changes in gut microbiota through the supplementation of a probiotic reduced cortisol concentration, and therefore acting as an anxiolytic (107). Changes in gut microbiota composition affect the production of cytokines and tryptophan in the host, which are the main two pathways involved in the behaviors of depression and anxiety (108). Nowadays, the influence of gut microbiota on behaviors have been demonstrated in rodents (23). However, studies in livestock remain uncommon and detailed effects of specific bacterial populations on behavioral traits are unknown. Even if studies on livestock are still uncommon, they have all implicated the gut microbiota in host behavior (109–113). In hens, the aggressive damaging behavior of feather pecking was associated higher relative abundances of *Clostridiales* and a lower relative abundance of *Lactobacillus* in the luminal microbiota composition of ileum, caecum and colon sections combined (114, 115). In humans, Parashar and Udayabanu (94) have reviewed the relationships between specific bacteria populations and both stress and anxiety. They showed that a rise in stress coincides mostly with an increase in *Bacteroidetes*, a decrease in *Actinobacteria, Firmicutes*, and *Proteobacteria*, whereas a rise in anxiety coincides with either an increase or a decrease in *Bacteroidetes* and *Firmicutes*, an increase in *Actinobacteria* or a decrease in *Proteobacteria*. In the current study, we also revealed that some indicators of aggression, responses to stress and exploration have medium to strong correlations with specific bacterial populations. The phyla identified were *Actinobacteria, Bacteroidetes, Fibrobacteres, Firmicutes, Proteobacteria*, and *Spirochaetes*. That means that any feed supplements that can promote their development may affect the behavioral response of its individuals. Indeed, the brain is shaped both directly and indirectly by the gut microbiota through several pathways involving metabolites and neurochemicals' secretions. These molecules reach the brain *via* the blood stream or the nervous system and affect its development, neural and pain processes, and modulation of the HPA axis, therefore modulating the behaviors of individuals (116).

## Conclusion

Comparisons between the three distinct 2-week diet strategies after transport on weaned pigs revealed that a short supplementation period can impact welfare indicators both during the administration period and after it. In terms of behaviors, GLN seemed to confer similar beneficial effects as antibiotic supplementation, and therefore should be considered as an alternative to the use of antibiotics. In terms of microbiota composition, supplementation produced significant shifts that disappeared over time. The effects of diet observed on behavioral indicators could be related to changes in microbiota composition as some specific bacterial taxa appeared to be associated with aggressiveness, stress and fear.

## Data Availability Statement

The data analyzed in this study can be accessed at https://www.ncbi.nlm.nih.gov/bioproject/PRJNA607434.

## Author's Note

Mention of trade names or commercial products in this article is solely for the purpose of providing specific information and does not imply recommendation or endorsement by the U.S. Department of Agriculture.

## Author Contributions

SP was responsible for study design, data collection, analysis and interpretation, and was the principle author of the manuscript. AD was responsible for study design, data collection and study coordination. BR was responsible for study conception and study coordination. SL was responsible for data analysis and interpretation. JJ was responsible for study design. JM-F was responsible for study design, data collection, analysis and interpretation, and was a major author of the manuscript. All authors contributed to manuscript revision and have read and approved the final manuscript.

### Conflict of Interest

The authors declare that the research was conducted in the absence of any commercial or financial relationships that could be construed as a potential conflict of interest.
